# Clinical Management and Prognostic Outcomes of Pancreatic Neuroendocrine Tumors: Insights from a Tertiary Care Center

**DOI:** 10.3390/medicina61111955

**Published:** 2025-10-30

**Authors:** Rucsandra-Ilinca Diculescu, Tudor Stroie, Doina Istrătescu, Adina Emilia Croitoru, Cristian Gheorghe, Vladislav Brașoveanu, Traian Dumitrașcu, Mihai Adrian Eftimie, Radu Țuțuian, Cătălina Poiană

**Affiliations:** 1 Faculty of Medicine, Carol Davila University of Medicine and Pharmacy, 050474 Bucharest, Romania; rucsandra-ilinca.diculescu@drd.umfcd.ro (R.-I.D.);; 2 Department of Gastroenterology, Fundeni Clinical Institute, 022328 Bucharest, Romania; 3 Department of Medical Oncology, Fundeni Clinical Institute, 022328 Bucharest, Romania; 4 Department of General Surgery, Fundeni Clinical Institute, 022328 Bucharest, Romania; 5 Faculty of Medicine, Berne University, 3012 Berne, Switzerland; 6 National Institute of Endocrinology, 011863 Bucharest, Romania

**Keywords:** pancreatic neuroendocrine tumors, surgery, overall survival, disease-free survival

## Abstract

*Background and Objectives:* Pancreatic neuroendocrine tumors (PanNETs) are rare and heterogeneous neoplasms with variable clinical behavior. Despite advances in diagnosis and treatment, the optimal management strategy remains unclear. This present study aims to evaluate survival outcomes according to first-line therapy and tumor characteristics. *Materials and Methods:* We conducted a retrospective cohort study including adult patients with histologically confirmed PanNETs who were treated at a tertiary care center between January 2020 and January 2025. Patients were divided into two groups according to their first-line management: surgical resection or non-surgical treatment. Overall survival (OS) was assessed in the entire cohort, and disease-free survival (DFS) was evaluated in patients who underwent complete surgical resection (R0). *Results:* A total of 68 patients were included, of whom 46 (67.6%) underwent surgery as first-line treatment. In the non-surgical group, 45.5% received combined systemic therapy and somatostatin analogues (SSA), 36.4% received SSA alone, 4.5% systemic therapy alone, and 9.1% were managed with active surveillance. Patients who underwent surgery tended to have lower-grade tumors and earlier-stage disease. The OS rates were 96.5% at one year, 83.2% at three years, and 62.3% at five years, with a median OS of 179 months. Both surgical treatment and lower tumor grade were significantly associated with improved OS. Among patients who had R0 resection, DFS was 95.8% at one year and 84.3% at both three and five years. Lower-grade tumors were also associated with longer DFS. *Conclusions:* This study demonstrates that first-line surgical resection and lower tumor grade were significantly associated with better OS in patients with PanNETs. Among patients who underwent curative surgery, lower tumor grades were associated with improved DFS.

## 1. Introduction

Neuroendocrine neoplasms (NENs) originate from neuroendocrine cells—rare cells that possess both endocrine and neuronal properties. These cells are located in endocrine organs and throughout the diffuse neuroendocrine system [1]. Approximately two-thirds of NENs arise in the digestive system, with the pancreas and small intestine among the most common primary sites [2].

Pancreatic neuroendocrine neoplasms (PanNENs) represent a small subset of pancreatic tumors, accounting for only 1–2% of cases. However, the pancreas is a frequent localization of these lesions, representing 31% of all neuroendocrine tumors (NETs) in a Portuguese multicenter study [3] and 23% in a Greek registry data [4] of all localizations [5]. The majority of these tumors (around 90%) are well-differentiated and classified as pancreatic neuroendocrine tumors (PanNETs), whereas poorly differentiated pancreatic neuroendocrine carcinomas (PanNECs) constitute a minority [6,7]. Based on presence or absence of hormonal syndrome about 70% of PanNETs are non-functioning pancreatic neuroendocrine tumors (NF-PanNETs) [6,8], manifesting no hormonal syndrome, often diagnosed at advanced stages and associated with significantly worse overall survival (OS), even following curative resection [9,10,11,12]. Functional PanNETs (F-PanNETs), which represent roughly 30% of cases, present with clinical hormonal syndrome and inadequate hormonal biochemical levels and are categorized based on their predominant hormone secretion, with insulinomas, gastrinomas, glucagonomas, somatostatinomas, and VIPomas being the most common subtypes [13,14].

Although NENs typically have a more favorable prognosis compared to many other malignancies, they constitute a highly heterogeneous group of tumors. Management strategies vary widely depending on tumor location, biological behavior, and patient-specific factors, making optimal therapeutic approaches a dynamic area of clinical research.

Although there is still no consensus on the definition of resectability status in NF-PanNETs, the ENETS 2023 guidelines aimed to clarify the primary treatment modalities for NF-PanNETs based on specific tumor characteristics [9]. Active surveillance can be considered in particular cases of tiny lesions (<10 mm) with no symptoms [9,15]. Lesions smaller than 20 mm may also be surveilled in elderly patients with significant comorbidities, especially when a duodenopancreatectomy would be required [15,16]. On the other hand, surgery should be recommended for younger patients without significant comorbidities or when signs of local invasion (such as main pancreatic duct dilation, jaundice, or suspected lymph node involvement) are present, requiring both pancreatectomy and lymphadenectomy [9,15,17].

Lesions measuring 10–20 mm represent a “grey zone”, for which the management should be individualized on a case-by-case basis [15,17,18].

For lesions larger than 20 mm and/or main pancreatic duct dilation, as well as in T3-T4 tumors, surgery with lymphadenectomy should be routinely performed [9]. Lesions of considerable size (>10 cm), lymph node involvement, and NF-Pan-NETs are associated with shorter survival after curative-intent resections [9]. Moreover, the decision to resect a primary PanNET in metastatic disease should also be individualized. Despite extrahepatic disease rarely causing death, primary tumor resection and hepatic cytoreductive surgery can improve survival and should be considered in selected patients [15,17].

In cases of advanced or unresectable functional tumors (F-PanNETs), apart from the control of tumor proliferation, the hormonal syndrome should also be addressed depending on the type of hormonal syndrome. For example, gastrinomas are treated with proton pump inhibitors (PPIs), somatostatin analogs (SSA) or peptide receptor radionuclide therapy (PRRT) in progressive cases. Insulinomas can be managed with dietary measures, diazoxide, PRRT, or Everolimus in advanced stages. A personalized approach is recommended, also considering the recently proven effectiveness of endoscopic ultrasound radio-frequency ablation (EUS-RFA), particularly useful in small insulinomas [9,19]. Moreover, in advanced stages of F-PanNETs, surgical resection—even when performed with a debulking intent—may offer greater clinical benefit compared to NF-PanNETs [15,17].

Several clinical guidelines have been developed by many professional organizations, such as the European Neuroendocrine Tumor Society (ENETS) and the North American Neuroendocrine Tumor Society (NANETS), alongside broader oncology bodies like NCCN, ASCO, and ESMO. However, there remains variability in their recommendations [20].

In Romania, a few studies have addressed clinicopathological and surgical prognostic markers in PanNETs; nevertheless, recent evidence on patients’ clinical profiles and treatment outcomes remains scarce [21,22,23].

The current study aims to provide a comprehensive analysis of PanNET cases managed in a tertiary care center, as well as to compare the outcomes of various first-line treatment strategies.

## 2. Materials and Methods

### 2.1. Study Design and Population

This retrospective study included all adult patients (≥18 years) diagnosed, evaluated, or treated for pancreatic neuroendocrine tumors (PanNETs) at the Fundeni Clinical Institute, a tertiary referral center in Bucharest, Romania, between January 2020 and January 2025. Patients with poorly differentiated pancreatic neuroendocrine carcinomas (PanNECs) or mixed neuroendocrine–non-neuroendocrine neoplasms (MiNENs) were excluded.

OS was defined as the time from diagnosis (or treatment initiation) to death from any cause, and disease-free survival (DFS) was defined as the time from surgery to the first recurrence or death. Survival probabilities were estimated using the Kaplan–Meier method, and differences between groups were assessed using the log-rank test. DFS analyses were conducted specifically in patients who underwent R0 resections to evaluate recurrence risk in this subgroup.

Recurrence was ascertained through review of patients’ electronic medical records, imaging reports and pathology results. Postoperative surveillance was performed according to European guidelines, adapted to national protocols and local institutional practice. It included contrast-enhanced CT or MRI, first performed 3–6 months after surgery. Subsequent imaging was performed every 6–12 months for the following 5 years, and then every 1–2 years for up to 10 years, or earlier if clinically indicated. Not all patients adhered to the scheduled follow-up, and some were lost to follow-up or continued follow-up at other institutions; these patients were consequently censored in the survival analysis.

### 2.2. Data Collection

Patient data were retrieved from institutional electronic medical records and outpatient documentation using ICD-10 codes corresponding to pancreatic lesions. Only cases with histopathological or cytological confirmation of PanNET were included. Collected data included demographics, tumor characteristics at diagnosis, details of perioperative and postoperative management, pathological findings, subsequent treatments, and follow-up information.

### 2.3. Classification and Grouping

Tumors were classified according to the 2022 World Health Organization (WHO) classification of neuroendocrine neoplasms. Patients were grouped based on first-line treatment: (1) surgical resection and (2) non-surgical therapy. All cases were managed in accordance with international clinical guidelines, incorporating multidisciplinary team (MDT) recommendations, patient preferences, and institutional expertise.

### 2.4. Statistical Analysis

Descriptive statistics were used to characterize the cohort. Categorical variables were compared using Fisher’s exact test, and continuous variables were analyzed with the Wilcoxon rank-sum test for non-normally distributed data. OS and DFS were estimated using the Kaplan–Meier method, with differences between survival curves assessed using the log-rank test. Statistical significance was set at *p* < 0.05. All analyses were performed using R software (version 4.5.1).

## 3. Results

### 3.1. Patient Characteristics

A total of 68 patients with histologically confirmed PanNETs were included. Of these, 46 patients (67.6%) underwent surgical resection as first-line treatment, while 22 (32.4%) received non-surgical therapies.

All cases were discussed in a multidisciplinary setting involving at least a surgeon, gastroenterologist, radiologist, and oncologist to determine the optimal treatment plan for each individual patient. Active surveillance was reserved for NF PanNETs < 1 cm without main pancreatic duct dilation, and for selected 1–2 cm lesions in patients declining surgery. Somatostatin analogs (SSA) were administered as Octreotide LAR 20–30 mg or Lanreotide Autogel 120 mg every 4 weeks, according to local protocol. Everolimus, Sunitinib, and Capecitabine-Temozolomide (CAPTEM) regimen were administered for patients with progressive advanced disease. PRRT was not available in our country during the study period; however, three patients from the surgical group and four from the non-surgical first-line group underwent PRRT treatment abroad in further treatment lines.

Among the non-surgical group, 45.5% received combined systemic therapy and SSA, 36.4% received SSA monotherapy, and 4.5% received systemic therapy alone. Active surveillance was employed in 9.1% of cases, and treatment details were unavailable for one patient (4.5%).

Baseline characteristics stratified by treatment group are presented in Table 1. Both groups were predominantly male, with similar median ages at diagnosis (56.5 vs. 53.5 years; *p* = 0.88). Most patients resided in urban areas and had good performance status (ECOG ≤ 2).

### 3.2. Tumor Characteristics

As shown in Table 2, tumors were most commonly located in the body or tail of the pancreas across both groups. Most tumors exceeded 20 mm in size, and the majority were classified as NET G1 or G2. However, NET G1 tumors were significantly more frequent in the surgical group (60.9% vs. 31.8%; *p* = 0.024), whereas NET G2 predominated in the non-surgical group. Only one NET G3 tumor was identified (surgical group). Lower TNM stages were significantly more common among surgically treated patients, while 72.7% of non-surgical patients presented with stage IV disease (*p* < 0.001). Metastases were more frequent in the non-surgical group (M1: 72.7% vs. 26.1%; *p* < 0.001). Functional tumors were more common in the non-surgical group (*p* = 0.01).

Table 3 provides more detailed information on tumor proliferation, including Ki-67 as both a continuous and categorical variable (per WHO 2022 grading) and the mitotic index.

### 3.3. Diagnostic Pathway

Referral pathways and diagnostic approaches are summarized in Table 4. Most surgical patients were directly referred to the surgical department (67.4%), while 50% of non-surgical patients were referred through gastroenterology (*p* < 0.001). Incidentally discovered tumors were common in both groups, but tumor-related symptoms were more often the presenting feature in surgically treated patients (56.5% vs. 9.1%; *p* < 0.001). CT imaging was the most frequently used diagnostic modality. Histological diagnosis was predominantly obtained from surgical specimens in the surgical group and via EUS-guided techniques or percutaneous liver biopsy in the non-surgical group (*p* < 0.001).

Endoscopic tissue acquisition was performed mainly by EUS-FNA, which represented the predominant and widely available technique during the early study period with sampling performed according to operator’s preference, while EUS-FNB was only sporadically adopted later. Detailed procedural variables such as needle type and gauge, number of passes, suction method, and rapid on-site evaluation (ROSE) availability were not recorded. However, ROSE is routinely carried out in our center, a specialized GI pathologist being available for EUS-FNA samples examination.

### 3.4. Surgical Treatment

The most common surgical procedure performed on the 46 patients was distal pancreatectomy (78.3%), followed by duodenopancreatectomy (10.9%) (Table 5). Synchronous liver metastasectomy was carried out in 28.2% of cases. The majority of interventions (91.3%) were performed using an open approach, and nearly three-quarters of patients were treated with curative intent. An R0 resection was achieved in 71.7% of patients (33 out of 34 patients treated with curative intent). Postoperative complications occurred in 24 patients (50% of the surgery cohort). According to the Clavien–Dindo classification, 14 patients (29.2%) experienced minor complications (Grades I–II), while 10 patients (20.8%) had major complications, including 7 (14.6%) Grade IIIa, 2 (4.2%) Grade IIIb, and 1 (2.1%) Grade IVa event. Most common complications were sterile fluid collections and postoperative pancreatic fistula (POPF) which occurred in 5/48 patients (10.4%). All cases required therapeutic drainage and were therefore classified according to the International Study Group of Pancreatic Surgery (ISGPS) as Grade B; no Grade C ISGPS events were recorded. Given the retrospective design, limited early documentation means a biochemical leak cannot be fully excluded in isolated cases, but an interventional approach justified Grade B classification. The majority of complications occurred among patients with open surgery, out of the 4 patients with laparoscopic surgery only one had a minor complication (fluid collection without need of reintervention).

Postoperative length of stay (LOS) data were available for 39 patients. The overall median LOS was 10 days (IQR 8–14). Data were missing for 7 patients due to incomplete records. Among the 4 patients who underwent laparoscopic distal pancreatectomy, the median LOS was 7 days (IQR 6–9), compared with 10 days (IQR 8–14) for those who had open surgery. Although LOS tended to be shorter after laparoscopic procedures, this difference was not statistically significant likely due to the small number of minimally invasive cases. No 30-day or 90-day mortality was observed.

### 3.5. Survival Outcomes

**Figure 1:**
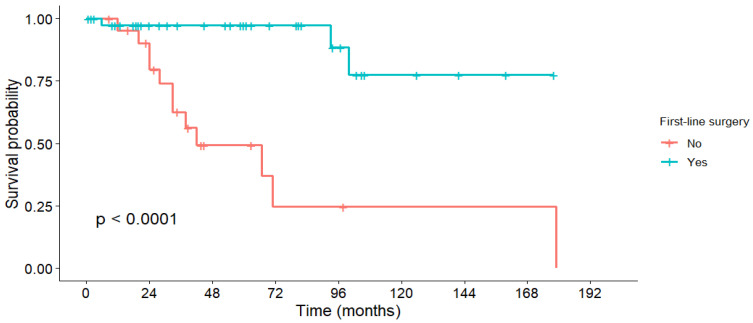
Overall survival stratified by first-line treatment.

**Figure 2:**
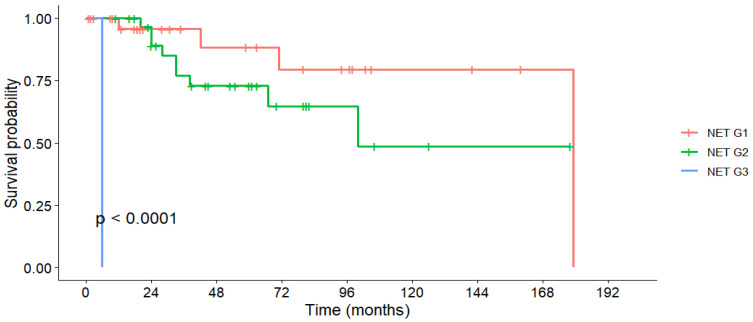
Overall survival stratified by tumor grading.

The overall cohort’s 1-, 3-, and 5-year OS rates were 96.5%, 83.2%, and 62.3%, with a median OS of 179 months (Table 6). First-line surgery resulted in significantly higher survival rates compared to non-surgical treatments (5-year OS: 97.3% vs. 49.3%; *p* < 0.001, Figure 1). Tumor grade was also strongly prognostic, with 5-year OS of 88.3% in G1 tumors and 72.8% in G2 tumors (*p* < 0.001, Figure 2). In contrast, lymph node invasion and distant metastases were associated with a numerically lower OS, but without statistical significance.

The sharp decline in survival curves for some subgroups at specific time points reflects the death of the final surviving patient in that group, a statistical artifact of the Kaplan–Meier method. This should not be interpreted as a sudden increase in death at a certain time point.

A multivariable Cox proportional hazards analysis including first-line surgery, tumor grade, and disease stage was performed. In this adjusted model, surgical resection remained strongly associated with improved OS (HR = 0.05, 95% CI 0.008–0.34, *p* = 0.002). Higher tumor grade was independently associated with increased risk of death (HR = 7.78, 95% CI 1.28–47.4, *p* = 0.026), whereas disease stage was not significantly related to survival in our analysis (HR = 1.02, 95% CI 0.54–1.96, *p* = 0.94).

While the multivariable Cox analysis suggests that the benefit of first-line surgery is independent of grade and stage, the results should be interpreted with caution due to the potential for overfitting (limited number of events—14 deaths).

### 3.6. Disease-Free Survival

**Figure 3:**
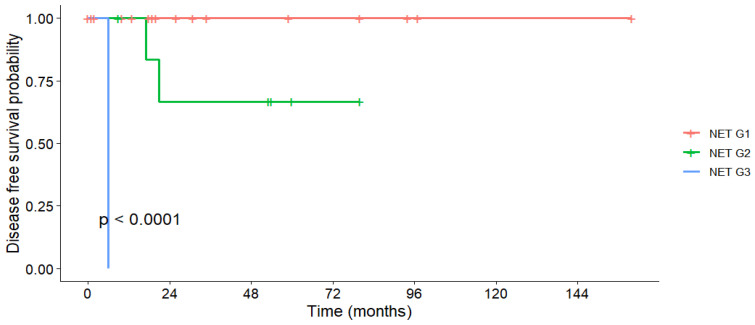
Disease-free survival stratified by tumor grading.

DFS for patients who underwent R0 resection (N = 33) was 95.8% at one year and 84.3% at three and five years, with no median DFS reached. Tumor grade was the only significant predictor: all G1 patients remained disease-free throughout follow-up, whereas one-third of G2 patients experienced recurrence within 5 years (*p* < 0.001, Figure 3). Tumor size, nodal status, and metastatic disease had no significant effect on DFS (Table 7).

No deaths occurred before recurrence in our cohort; therefore, all DFS events correspond to radiologically or pathologically confirmed recurrences.

## 4. Discussion

Given the rarity of PanNETs and limited local data from Romanian tertiary care centers, our findings provide valuable insight into patient characteristics, treatment patterns, and clinical outcomes in this setting.

The median age at diagnosis was similar in both groups (56.5 years in the surgical group vs. 53.5 years in the non-surgical group), aligning with the existing literature that describes PanNET onset typically in the fifth or sixth decade of life [24,25,26,27,28].

As expected, patients undergoing surgery were more frequently diagnosed due to tumor-related symptoms, with initial referrals often made directly to surgical departments (67.4%). In contrast, non-surgical cases were commonly evaluated in gastroenterology, highlighting the need for interdisciplinary coordination in PanNET management.

EUS-FNA often performed with on-site pathological assessment, was more commonly employed than EUS-FNB during the study period, reflecting practice patterns prior to widespread adoption of FNB, despite its now-proven diagnostic superiority [29].

Over one-third of surgical patients were asymptomatic at diagnosis, consistent with the increasing detection of incidentalomas. In our cohort, 39.1% of tumors were ≤20 mm and 65.2% were non-functional, in line with the literature data [9,10,11]. Notably, 18.2% of small tumors (<20 mm) were in the non-surgical group, likely reflecting clinical judgment based on tumor biology, comorbidities, or the unresectability of the tumors.

A growing body of evidence supports the use of active surveillance for small, low-grade, non-functional PanNETs. Studies such as the PANDORA and ASPEN trials are currently exploring the safety and outcomes of non-operative strategies in tumors < 2 cm [9,30,31]. However, surgical intervention may still be appropriate for selected small lesions, as there is no clear size threshold that reliably excludes malignancy [32,33,34].

In our cohort, 65.2% of surgically treated patients were node-negative (N0), suggesting early-stage presentation. Node-positive patients (N1) demonstrated reduced 3- and 5-year OS, consistent with previous studies that have linked nodal involvement with recurrence and poorer prognosis [35].

Curative-intent surgery was performed in 34 patients, with R0 resection achieved in 97.1% (33/34). One-third of R0 patients required no further treatment postoperatively, underlining the potential for long-term disease control following complete resection. While loss to follow-up limits full interpretation, it may also reflect clinical remission in this subgroup. Surgery for small, low-risk PanNETs is still a subject of debate. Some authors advocate for resection of all non-functional tumors due to their malignant potential, even in lesions under 2 cm [32,33,34]. In contrast, others report favorable outcomes with surveillance, particularly in incidentally discovered G1 tumors [32,36,37]. In our cohort, 18 small tumors underwent surgical resection. Of the four that did not, three were G2 tumors; one patient later required surgery due to progression, and another progressed to unresectable disease requiring systemic therapy.

Consistent with tumor location, distal pancreatectomy was the most commonly performed procedure. Four were completed laparoscopically, reflecting international guideline recommendations favoring minimally invasive surgery for body-tail lesions to reduce morbidity [9,38]. Over time, improved surgical technique and perioperative care may have contributed to lower complication rates, as also observed in a recent Romanian study reporting no 30-day mortality among 16 surgically managed PanNETs [21].

The OS in our cohort was 96.5% at 1 year, 83.2% at 3 years, and 62.3% at 5 years, with a median OS of 179 months—slightly more favorable than in comparable cohort studies [39,40]. Although most tumors were larger than 20 mm, smaller lesions were associated with better outcomes. Other studies support the benefit of resecting small tumors, though active surveillance remains a valid option in low-risk cases [25,41].

Concerns regarding overtreatment have prompted the reevaluation of surgical indications. Partelli et al. found that one-third of resected patients had no aggressive features and remained disease-free post-R0 resection, suggesting possible overtreatment [42]. In our study, several small G2 tumors were managed non-operatively, with variable outcomes depending on progression and comorbidities. These cases underline the importance of individualized treatment planning.

Predictive factors for surgery in recent prospective studies included tumor size, ductal dilation, BMI, patient age, and center expertise, though only a minority of surgeries were performed due to high-risk features such as duct dilation or metastases [31]. Additionally, age alone does not appear to predict tumor aggressiveness, cautioning against excessive surgical intervention in younger patients [31,43].

DFS in patients with R0 resection was excellent, with rates of 95.8% at 1 year and 84.3% at both 3 and 5 years. While our results are comparable to some series [44], others report slightly lower DFS rates [45]. In our study, DFS was superior in lower-grade tumors and smaller lesions, consistent with other reports identifying grade, size, and Ki-67 index as key prognostic factors [9,12,34]. Interestingly, patients with metastatic disease who underwent resection of both primary and metastatic sites showed encouraging short-term DFS (100% at 1 year), though longer-term outcomes remain uncertain. The literature on simultaneous resection remains limited, but suggests potential survival benefit in selected patients [46].

Tumor grade strongly influenced prognosis. In our cohort, 5-year OS was 88.3% for G1 and 72.8% for G2. The sole G3 patient died early, precluding broader conclusions. Comparable multicenter studies report 5-year OS rates of 95%, 82%, and 35% for G1, G2, and G3 tumors, respectively [47]. Some reports, however, have found no OS differences between G1 and G2 tumors [25].

Overall, our findings support the role of first-line surgery in eligible PanNET patients, particularly those with localized, low-grade tumors. However, the decision between surgery and surveillance remains nuanced, requiring further prospective studies.

Moreover, it should be acknowledged that the improved survival observed among surgically treated patients may not solely reflect the effect of surgery, but also the selection of patients with earlier-stage, biologically indolent disease and favorable clinical features.

### Strengths and Limitations

This retrospective study was conducted in a tertiary referral center with a multidisciplinary approach involving surgery, gastroenterology, oncology, pathology, endocrinology and radiology teams. It provides comprehensive, real-world data on the diagnosis, management, and outcomes of patients with pancreatic neuroendocrine tumors from a population of patients that is underrepresented in the international literature. Although there is currently no national database of patients with PanNETs, this study represents an important step toward developing such a registry and strengthening the national framework for specialized care. The consistent institutional protocols, specialized expertise, and integrated decision-making processes contribute to the reliability and clinical relevance of the findings, helping to identify prognostic factors and treatment patterns applicable to everyday practice.

However, the study has several limitations. Its retrospective design and modest sample size (N = 68), though reflective of PanNET’s low incidence (0.33–0.94 per 100,000) [5,48,49], limit generalizability. A key methodological limitation is the non-randomized comparison between surgically and non-surgically treated patients: the choice between surgical and non-surgical management was influenced by multiple clinical factors, including tumor size, grade, location, presence of metastases, and patient comorbidities, which further complicates direct comparison of outcomes between groups. Selection bias is possible, as surgically treated patients may have had better performance status or more localized disease. Additionally, the unavailability of advanced imaging modalities such as ^68^Ga-DOTA PET/CT and PRRT in Romania may have affected staging accuracy and treatment options. Follow-up data were also limited in some cases, complicating long-term outcome assessment.

Efforts are currently underway by the Romanian NENs Association to establish a certified multidisciplinary center, which will enable more standardized and comprehensive care for PanNET patients.

## 5. Conclusions

This study highlights the central role of surgery in the management of PanNETs, offering real-world insights from a Romanian cohort treated at a high-volume, multidisciplinary tertiary center. Our findings align with the existing literature, demonstrating that patients with resectable PanNETs who undergo first-line surgery and those with lower tumor grades experience significantly better OS. Among patients who achieved R0 resections, lower tumor grades were also associated with improved DFS.

However, the favorable evolution of patients that underwent surgery should be regarded in the whole clinical context, acknowledging the fact that this could be not only due to the surgical intervention, but also to the patients’ selection.

These results underscore the importance of early diagnosis, accurate tumor grading, and timely surgical intervention. However, further prospective studies with larger cohorts and longer follow-up are needed to refine therapeutic strategies and long-term surveillance protocols in this heterogeneous and rare disease population.

## Figures and Tables

**Table 1:** Characteristics of PanNET patients.

Characteristic	First-Line Surgery		*p*-Value
	YES	NO	
**Male gender, *n*** (%)	26 (56.5)	15 (68.2)	0.43
**Median age at diagnosis,** *years* (IQR)	56.5 (43.75–65)	53.5 (51.25–62.75)	0.88
**Urban area,** *n* (%)	31 (67.4)	15 (68.2)	1
**Follow-up,** *median* (IQR)	40 (10.25–81.75)	34 (24–44.75)	0.96
**Other significant comorbidities,** *n* (%)	34 (73.9)	13 (59.1)	0.58
**Performance status at diagnosis,** *n* (%)			
- ECOG 0	18 (39.1)	7 (31.8)	0.72
- ECOG 1	27 (58.7)	14 (63.6)	
- ECOG 2	1 (2.2)	1 (4.5)	
- ECOG > 2	0 (0)	0 (0)	

**Table 2:** Tumor-related characteristics.

Characteristic		First-Line Surgery		*p*-Value
		YES	NO	
		N (%)	N (%)	
**Location**	Head	10 (21.7)	6 (27.3)	0.52
	Body and tail	36 (78.3)	12 (54.5)	
	Missing	0 (0)	3 (13.6)	
**Size**	≤20 mm	18 (39.1)	4 (18.2)	0.15
	>20 mm	24 (52.2)	14 (63.6)	
	Missing	4 (8.7)	4 (18.2)	
**Tumor grading**	NET G1	28 (60.9)	7 (31.8)	0.024
	NET G2	16 (34.8)	15 (68.2)	
	NET G3	1 (2.2)	0 (0)	
	Missing	1 (2.2)	0 (0)	
**T stage**	T1	17 (37)	0 (0)	0.012
	T2	9 (19.6)	4 (18.2)	
	T3	7 (15.2)	1 (4.5)	
	T4	5 (10.9)	4 (18.2)	
	Missing	8 (17.4)	13 (59.1)	
**N stage**	N0	30 (65.2)	5 (22.7)	0.004
	N1	6 (13)	8 (36.4)	
	Missing	10 (21.7)	9 (40.9)	
**M stage**	M0	32 (69.6)	6 (27.3)	<0.001
	M1	12 (26.1)	16 (72.7)	
**Site of metastases**	Liver	10 (21.7)	11 (50)	0.66
	Extrahepatic	1 (2.2)	1 (4.5)	
	Both	1 (2.2)	4 (18.2)	
**TNM stage at diagnosis**	Stage 1	20 (43.5)	0 (0)	<0.001
	Stage 2	7 (15.2)	3 (13.6)	
	Stage 3	6 (13)	3 (13.6)	
	Stage 4	12 (26.1)	16 (72.7)	
	Missing	1 (2.2)	0 (0)	
**Tumor functional status**	Non-functional	30 (65.2)	10 (45.5)	0.01
	Carcinoid syndrome	4 (8.7)	10 (45.5)	
	Gastrinoma	1 (2.2)	0 (0)	
	Missing	11 (23.9)	2 (9.1)	

**Table 3:** Detailed information on tumor proliferation.

Tumor Grade	First-Line Surgery (n = 46)			Non-Surgery (n = 22)		
	n (%)	Ki-67 (%) Median, IQR	Mitotic Index * Median, IQR	n (%)	Ki-67 (%) Median, IQR	Mitotic Index * Median, IQR
G1	28 (60.9)	2 (1–2)	1 (0–1)	7 (31.8)	2 (2–2)	1 (0–1)
G2	16 (34.8)	7 (5–10)	3 (2–5)	15 (68.2)	6 (5–10)	3 (2–6)
G3	1 (2.2)	40 (40)	15	0	-	-
Missing	1 (2.2)	-	-	0	-	-

* mitoses/10 High-Power Field (HPF).

**Table 4:** Diagnosis of PanNETs.

		First-Line Surgery		*p*-Value
		YES N (%)	NO N (%)	
**Department of referral**	Gastroenterology	14 (30.4)	11 (50)	<0.001
	Surgery	31 (67.4)	5 (22.7)	
	Oncology	1 (2.2)	5 (22.7)	
	Internal medicine	0 (0)	1 (4.5)	
**Mode of diagnosis**	Incidentaloma	15 (32.6)	14 (63.6)	<0.001
	Functional syndrome	1 (2.2)	1 (4.5)	
	Tumor-related symptoms	26 (56.5)	2 (9.1)	
	Screening programs	2 (4.3)	3 (13.6)	
	Missing	2 (4.3)	2 (9.1)	
**Imaging procedures**	CT scan	22 (47.8)	13 (59.1)	0.72
	MRI	12 (26.1)	3 (13.6)	
	Multiple	10 (21.7)	5 (22.7)	
	Missing	2 (4.3)	1 (4.5)	
**Histopathology sample collection**	EUS with FNA	12 (26.1)	9 (40.9)	<0.001
	EUS with FNB	2 (4.3)	3 (13.6)	
	Surgery	32 (69.6)	3 (13.6)	
	Percutaneous liver biopsy	0 (0)	6 (27.3)	
	Missing	0 (0)	1 (4.5)	
**Source of histopathology sample**	Primary tumor	39 (84.8)	11 (50)	0.001
	Lymph nodes	1 (2.2)	0 (0)	
	Metastases	2 (4.3)	8 (36.4)	
	Multiple	4 (8.7)	2 (9.1)	
	Missing	0 (0)	1 (4.5)	

**Table 5:** Surgery.

Surgery	N (%)
Type of surgical procedure	
Duodenopancreatectomy	5 (10.9)
Distal pancreatectomy	36 (78.3)
Segmental pancreatectomy	1 (2.2)
Enucleation	2 (4.3)
Total pancreatectomy	1 (2.2)
Debulking	1 (2.2)
Synchronous liver metastasectomy	13 (28.2)
Curative intent	34 (73.9)
R0 resection	33 (71.7)

**Table 6:** Overall survival in the entire cohort.

Stratification	Group	1-Year OS	3-Year OS	5-Year OS	Median OS (Months)	Mean OS (Months)	*p*-Value
**Gender**	Male	94.6%	87.8%	80.3%	100	119.4	0.76
	Female	100%	73.9%	73.9%	179	138.5	
**Lymph node invasion (N1)**	Yes	91.7%	67.9%	54.3%	-	100.5	0.2
	No	96.2%	91.3%	91.3%	-	122.1	
**Metastatic disease (M1)**	Yes	96.2%	76.3%	70.9%	179	117.9	0.39
	No	96.8%	89%	84.1%	-	130.9	
**Tumor Grading**	Grade 1	95.7%	95.7%	88.3%	179	152.1	<0.001
	Grade 2	100%	76.9%	72.8%	100	116.3	
	Grade 3	0%	0%	0%	6	6	
**Functional syndrome**	Yes	93.3%	65.3%	65.3%	71	94.5	0.14
	No	100%	94.1%	79.6%	-	137.2	
**First-line surgery**	Yes	97.3%	97.3%	97.3%	-	158	<0.001
	No	95.2%	62.6%	49.3%	42	75.9	

**Table 7:** Disease-free survival in R0 resected patients.

Stratification	Group	1-Year DFS	3-Year DFS	5-Year DFS	Median DFS (Months)	Mean DFS (Months)	*p*-Value
**Location**	Head	100%	100%	100%	-	160	0.37
	Body and tail	94.7%	80.4%	80.4%	-	131.7	
**Size**	≤20 mm	100%	100%	100%	-	160	0.12
	>20 mm	91.7%	78.6%	78.6%	-	128.4	
**Lymph node invasion (N1)**	Yes	100%	100%	100%	-	160	0.69
	No	95.2%	89.6%	89.6%	-	144.7	
**Metastatic disease (M1)**	Yes	100%	66.7%	66.7%	-	112.3	0.3
	No	95%	87.7%	87.7%	-	142.1	
**Tumor Grading**	Grade 1	100%	100%	100%	-	160	<0.001
	Grade 2	100%	66.7%	66.7%	-	113	
	Grade 3	0%	0%	0%	6	6	

## Data Availability

The data that support the findings of this study are available from the corresponding author, upon reasonable request.
